# Intermittent Fevers

**Published:** 1844-11-16

**Authors:** 


INTERMITTENT FEVERS.

Dr. Crawford states, in the Montreal Medical Gazette, that intermittent and remittent fevers are extremely prevalent in Upper Canada, whilst they are very rare in the lower division of the province. An extraordinary exemption from this class of fevers is also observable in Nova Scotia, and New Brunswick. At Annapolis, Windsor, and Fort Cumberland, in Nova Scotia, which are situated at the embouchures of rivers, daily exposed to extensive inundations by the rise of the tide, where the banks for several miles exhibit a combinatin of mud, marsh, and decayed vegetation, so generally considered a prolific source of this class of febrile diseases—intermittents and remittents are extremely rare; and at Fredericktown, in New Brunswick, situated on the marshy banks of a river, surrounded by a dense wood and luxuriant vegetation, these diseases are scarcely ever met with; proving that, although, on some occasions, circumstances of this nature may favour the development of this disease, its prevalence or existence is by no means a necessary consequence or concomitant; while, on the other hand, this class of diseases has been very prevalent at Gibraltar, the Ionian Islands, and Bermuda, countries which are destitute of marsh, and comparatively barren of vegetation.—*Ibid.*

